# The Preoperative Factors for the Undercorrection of Myopia in an Extend Depth-of-Focus Intraocular Lens: A Case-Control Study

**DOI:** 10.3390/diagnostics14141499

**Published:** 2024-07-12

**Authors:** Chia-Yi Lee, Hung-Chi Chen, Ie-Bin Lian, Chin-Te Huang, Jing-Yang Huang, Shun-Fa Yang, Chao-Kai Chang

**Affiliations:** 1 Institute of Medicine, Chung Shan Medical University, Taichung 40201, Taiwan; 2 Nobel Eye Institute, Taipei 10041, Taiwan; 3 Department of Ophthalmology, Jen-Ai Hospital Dali Branch, Taichung 41265, Taiwan; 4 Department of Ophthalmology, Chang Gung Memorial Hospital, Linkou, Taoyuan 33305, Taiwan; 5 Center for Tissue Engineering, Chang Gung Memorial Hospital, Linkou, Taoyuan 33305, Taiwan; 6 Department of Medicine, Chang Gung University College of Medicine, Taoyuan 33305, Taiwan; 7 Institute of Statistical and Information Science, National Changhua University of Education, Chunghua 50007, Taiwan; 8 Department of Ophthalmology, Chung Shan Medical University Hospital, Taichung 40201, Taiwan; 9 Department of Ophthalmology, School of Medicine, Chung Shan Medical University, Taichung 40201, Taiwan; 10 Department of Medical Research, Chung Shan Medical University Hospital, Taichung 40201, Taiwan; 11 Department of Optometry, Da-Yeh University, Chunghua 51591, Taiwan

**Keywords:** extend depth-of-focus, spherical equivalent, axial length, total corneal refractive power, myopia

## Abstract

We aim to investigate the potential risk factors for undercorrection in those who have received extend depth-of-focus (EDOF) intraocular lens (IOL) implantation. A retrospective case-control study was conducted in which patients who had received one type of EDOF IOL implantation were included. The patients were divided into the residual group and non-residual group according to the final postoperative sphere power. The preoperative data include the refractive, topographic, endothelial, and biometric parameters obtained. A generalized linear model was generated to yield the adjusted odds ratio (aOR) and 95% confidence interval (CI) of each parameter of the residual myopia. One month postoperatively, the UDVA was better in the non-residual group than in the residual group (*p* = 0.010), and the final SE was significantly higher in the residual group than in the non-residual group (*p* < 0.001). In the multivariable analysis, the high preoperative cycloplegia sphere power, higher TCRP, higher corneal cylinder power, and longer AXL significantly correlated to the presence of postoperative residual myopia (all *p* < 0.05). Furthermore, the higher preoperative cycloplegia sphere power, higher TCRP, higher corneal cylinder power, longer AXL, larger ACD, and larger WTW were significantly associated with postoperative residual myopia in the high-myopia population (all *p* < 0.001), while the higher preoperative cycloplegia sphere power, higher TCRP, and longer AXL were related to postoperative residual myopia in the low-myopia population (all *p* < 0.05). In conclusion, high preoperative myopia and corneal refractive power correlate to high risk of residual myopia after EDOF IOL implantation, especially in the high-myopia population.

## 1. Introduction

The cataract is a common ophthalmic disease which results in the visual impairment of nearly 90 million persons in the world [1]. The clinical presentation of cataract include progressively reduced vision, monocular diplopia, membranous sensation, reduced near vision, and glare [1,2]. The only effective and credible method to manage the impaired vision resulting from a cataract is the execution of cataract surgery [3]. Generally, postoperative visual acuity will recover after uneventful cataract surgery [4,5], and several types of intraocular lens (IOL) can be applied to reach an acceptable far or near visual acuity [6,7].

The extend depth-of-focus (EDOF) IOL is a presbyopia-correcting IOL which has been utilized since the early 2000 and remains popular today [8,9]. The average postoperative uncorrected distance visual acuity (UDVA) after the EDOF IOL implantation has been determined to be about 0.10 LogMAR in preceding research [10]. Furthermoe, the refractive status after cataract surgery was within expectations in those with EDOF IOL implantation for both the emmetropia target and monovision target [11]. Nevertheless, some postoperative complications observed in individuals who had received EDOF IOL implantation include poor UDVA, glare, persistent hale, and starburst, although the incidence of these was relatively low [12]. In addition, postoperative residual myopia is another complication that can significantly reduce the postoperative vision, and IOL exchange may be needed to manage it [13].

Some risk factors associated with postoperative residual myopia after cataract surgery have been found. High preoperative myopia contributes to the prominent residual sphere power after cataract surgery [14]. Additionally, corneal curvature relates to a higher incidence of postoperative residual astigmatism, which warrants additional adjustment for toric IOL [15]. Still, it remains unclear whether preoperative factors could influence postoperative residual myopia in those with EDOF IOL implantation. Moreover, since preoperative myopia has been correlated with a higher chance of postoperative refractive instability [16], the risk factor for postoperative residual myopia may be different in patients with different degrees of preoperative myopia, which thus require investigation.

Accordingly, the purpose of the present study is to evaluate the potential risk factors for postoperative residual myopia in patients with one type of EDOF IOL implantation. The risk factors for postoperative residual myopia in connection with different levels of preoperative myopia were also analyzed.

## 2. Materials and Methods

### 2.1. Participant Selection

A retrospective case-control study was executed in the Nobel Eye Institute, which has multiple clinics in the central, southern, and northern areas of Taiwan. Participants were selected for the present study if they (1) were aged between 50 and 100 years, (2) had received a complicated or senile cataract diagnosis at the Nobel Eye Institute, (3) had received cataract surgery as well as EDOF IOL implantation at the Nobel Eye Institute, and (4) had been followed in any branch of Nobel Eye Institute for at least one month. On the other hand, successive exclusion criteria were used to omit participants with the following special statuses: (1) a preoperative corrected distance visual acuity (CDVA) worse than hand motion, (2) a diagnosis of earlier eyeball rupture event, (3) a diagnosis of central corneal opacity or earlier central-involved microbial keratitis, (4) a diagnosis of prominent retinal disease such as macula-involved rhegmatogenous retinal detachment or vitreous hemorrhage, (5) a diagnosis of end-stage glaucoma, (6) a diagnosis of ischemic optic neuropathy, or (7) the receipt of monovision (planned residual myopia) intervention in any eye. Notably, patients with previous refractive surgery were not excluded from the present study. In the next step, the participants were categorized into the residual myopia group and non-residual myopia group according to their postoperative myopia status. The definition of residual myopia was set as a sphere power greater than −0.75 diopter (D) one month after the cataract surgery. Only the first eye that received cataract surgery was enrolled in the present study, and one eye with residual myopia was matched to five eyes that did not show residual myopia and received EDOF IOL implantation within the same two months. After the whole selection process, a total of 42 eyes from 42 participants were included, and 35 and 7 eyes were placed in the non-residual group and residual group, respectively.

### 2.2. Surgical Details

All the cataract surgeries in the present study were done by one experienced cataract specialist (C.-Y.L.), and one phacoemulsification device (Centurion, Alcon, Fort Worth, TX, USA) was utilized for all cataract surgeries. IOL power was calculated with the Barrett formula due to its high precision in an earlier study [17]. The main incision was done by superior-approach method, and the ophthalmic viscoelastic device was injected into the anterior chamber. After making the continuous curvilinear capsulorhexis, the hydrodissection procedure was performed before side-port creation. The phaco-chop technique was employed to clean the nucleus fragments, and remaining cortex was removed by an infusion–aspiration probe. One type of EDOF IOL (AcrySof^®^ IQ Vivity^®^, Alcon, Fort Worth, TX, USA) was implanted into the capsular bag, and the retained ophthalmic viscoelastic device was extracted by the same infusion–aspiration probe. A hydroseal technique was employed to obstruct the main corneal incision and side-port. Finally, an ointment containing 0.3% tobramycin and 0.1% dexamethasone (Tobradex Sterile Ophthalmic Ointment, s.a. Alcon-Couvreur n.v., Rijksweg, Puurs, Belgium) was dropped onto the ocular surface. In the postoperative period, levofloxacin, prednisolone, and tobradex were employed for about 7 days, after which these agents were replaced by dexamethasone/neomycin agents for about 7 days. Then, sulfamethoxazole and fluorometholone agents were applied for about three weeks.

### 2.3. Ophthalmic Examinations

All the participants in the present study received the same protocol of preoperative examinations at the Nobel Eye Institute. The preoperative exams involve UDVA and CDVA measurement, cycloplegic refraction of both sphere power and cylinder power by autorefractor (KR-8900, Topcon, Itabashi-ku, Tokyo, Japan), and intraocular pressure (IOP) measurement by pneumatic tonometry (NT-530, Nidek Co., Ltd., Gamagori, Japan). Central corneal thickness (CCT), steep and flat keratometry (K), total corneal refractive power (TCRP), corneal cylinder power, angle kappa, pupil diameter, higher-order aberrations (HOA), and spherical aberration (SA) were obtained by a topographic machine (TMS-5, Tomey Corporation, Nishi-Ku, Nagoya, Japan). Furthermore, the axial length (AXL), anterior chamber depth (ACD), lens thickness (LT), and corneal diameter presented as white-to-white (WTW) were obtained via biometry machine (IOL Master 700, Carl Zeiss, Göschwitzer Str., Jena, Germany). Finally, the endothelial cell density (ECD), the coefficient of variant (CV), and the hexagonality (HEX) were recorded using a specular microscope (CEM-530, Nidek Co., Ltd., Gamagori, Japan). The postoperative exams involve the UDVA, uncorrected near visual acuity (UNVA), IOP, manifest sphere power, and cylinder power. The postoperative exams used exactly the preoperative devices. Ophthalmic exams were taken before, one week after, two weeks after, and one month after the cataract surgery. The spherical equivalent (SE) in the present study was set as sphere power plus half of the cylinder power.

### 2.4. Statistical Analysis

The SPSS version 20.0 (SPSS Inc., Chicago, IL, USA) was applied for statistical analysis in this study. The statistical power of the current study was 0.67 with a 0.05 alpha value and a medium effect size which was generated using the G*power version 3.1.9.2 (Heinrich Heine Universität at Düsseldorf, Germany). The Shapiro–Wilk test was applied to check the normality of data in our study population and displayed abnormal distribution (all *p* < 0.05). Descriptive analysis was applied to present the age, sex, pre-existing diseases, earlier ocular surgeries, UDVA, CDVA, preoperative myopia, preoperative astigmatism, topographic factors, endothelial covariates, biometric values, and postoperative outcomes. The Mann–Whitney U test and Fisher’s exact test were applied to analyze the preoperative and postoperative characteristics between the non-residual group and residual group. A generalized linear model was then applied to evaluate the significant risk factor for postoperative residual myopia between the two groups after adjusting for age and sex. The adjusted odds ratio (aOR) with correlated 95% confidence interval (CI) for prominent residual myopia was produced by the generalized linear model. For the subgroup analysis, the participants were divided into a high-myopia subgroup and low-myopia subgroup according to whether the preoperative cycloplegia SE exceeded −6.00 D, according to a previous report [18], and the generalized linear model was applied again to evaluate the risk factors for prominent residual myopia in the different subgroups. A *p* value lower than 0.05 was regarded as statistical significance, and a *p* value less than 0.001 was depicted as *p* < 0.001.

## 3. Results

The preoperative characteristics of the two groups are depicted in Table 1. The mean age was 59.00 ± 9.98 years in the non-residual group and 56.20 ± 12.33 years in the residual group, and the differences of age between the two groups were insignificant (*p* = 0.699). The sex distributions between the two groups were also statistically identical (*p* = 0.580). As for the preoperative parameters, all refractive, topographic, biometric, and endothelial parameters demonstrated non-significant differences between the non-residual and residual groups (all *p* > 0.05) (Table 1).

One day after the cataract surgery, the UDVA and UNVA were statistically similar between the non-residual and residual group (*p* = 0.099 and 0.053, respectively), and the SE showed a significantly higher value in the residual group than in the non-residual group (−1.10 ± 0.74 versus −0.39 ± 0.30, *p* = 0.004) (Table 2). One month postoperatively, the UDVA was better in the non-residual group than in the residual group (0.07 ± 0.07 versus 0.22 ± 0.19, *p* = 0.010), while the UNVA between the two groups showed an insignificant difference (*p* = 0.125). The final SE was still significantly higher in the residual group than in the non-residual group (−1.12 ± 0.57 versus −0.21 ± 0.35, *p* < 0.001) (Table 2). In the multivariable analysis, the high preoperative cycloplegia sphere power, higher TCRP, higher corneal cylinder power, and longer AXL significantly correlated to the presence of postoperative residual myopia (all *p* < 0.05) (Table 3).

In the subgroup analysis stratified by the degree of preoperative myopia, higher preoperative cycloplegia sphere power, higher TCRP, higher corneal cylinder power, longer AXL, larger ACD, and larger WTW were significantly associated with the presence of postoperative residual myopia in the high-myopia population (all *p* < 0.001) (Table 4). On the other hand, higher preoperative cycloplegia sphere power, higher TCRP, and longer AXL related to the presence of postoperative residual myopia in the low-myopia population (all *p* < 0.05) (Table 5).

## 4. Discussion

In the present study, the postoperative UDVA and SE were significantly worse in the residual group than in the non-residual group. The presence of higher preoperative myopia, higher TCRP, and higher corneal cylinder power occurred more frequently in the residual group than in the non-residual group. In addition, greater ACD and WTW were also present in the residual group more frequently in the high-myopia population.

For all the participants that received EDOF IOL implantation, higher preoperative myopia and higher corneal refractive status were associated with postoperative residual myopia. In the previous study, high preoperative myopia was associated with higher incidence of undercorrection in the individuals who had received cataract surgery [14]. Furthermore, high myopia status also correlated to a higher degree of postoperative residual myopia in the case of corneal refractive surgery [19]. Nevertheless, the possible preoperative factors for postoperative residual myopia after EDOF IOL implantation had not been fully explored. To our knowledge, this may be a preliminary study to display the possible risk factors of postoperative residual myopia after EDOF IOL implantation. Moreover, all the cataract surgeries were completed by one ophthalmologist, thus the confounding effect of surgical technique on postoperative outcomes could be minimal. On the other side, we adjusted for age and sex in the generalized linear model to decrease the effect of these two confounding factors on analysis. Consequently, the high myopic status and higher corneal refractive power could be credible parameters for postoperative residual myopia in those who have received EDOF IOL implantation. A possible reason for the correlation between high preoperative myopia and postoperative residual myopia may be that high AXL is associated with a large capsular bag and subsequent IOL movement [20,21,22], and the final position of the IOL may not be located at the exact site we scheduled, which could contribute to myopia shift. In addition, high AXL could diminish the IOL calculation precision, and the undercorrection may occur more frequently under such conditions [23]. As for the corneal aspect, high corneal curvature was associated with higher incidence of postoperative residual astigmatism in general cataract surgery [24], and high corneal curvature can also reduce the predictability of small-incision lenticule extraction [19]. Also, previous experience has shown that high corneal astigmatism can cause interference of the IOL measurement [25]. As a consequence, it is reasonable for the TCRP and corneal cylinder power to influence the risk of undercorrection in EDOF IOL implantation.

In the subgroup analysis considering the degree of preoperative myopia, high preoperative myopia, high AXL, high TCRP, high corneal cylinder power, high ACD, and high WTW correlated to a higher risk of postoperative residual myopia in the high-myopia population. The previous study showed that high myopia can contribute to the higher variation of postoperative refractive status after cataract surgery [16], and secondary management for refractive correction may be advocated for those high-myopia patients with prominent post-cataract surgery refractive error [26]. In the high-myopia subgroup, large ACD and large WTW were associated with postoperative residual myopia, in addition to the risk factors in the general population. There was scant research to demonstrate this correlation. The ACD is an important parameter in the calculation of IOL power, though its influence is not as prominent as that of the AXL [27]. We speculate that the ACD would significantly influence the IOL calculation for the EDOF IOL only in specific conditions, including high AXL/high myopia. Moreover, large WTW also related to higher risk of undercorrection in the high-myopia subgroup of the present study. The WTW is also a parameter for IOL calculation in the recent IOL formula, although it plays a minor role [28], and greater WTW was associated with longer lens diameter and possibly a larger capsular bag [29]. Thus, WTW may influence the rate of overcorrection in specific conditions, similar to the ACD. However, the significance of WTW in the analysis was only marginal, and further study, with higher case numbers, is needed to clarify the exact correlation between WTW and undercorrection in EDOF IOL. In the low-myopia subgroup, the corneal cylinder power did not serve as a risk factor for overcorrection. It may be that the reduction of AXL led to a higher predictability of refraction and the effect of corneal cylinder power was hidden to some degree.

With respect to the postoperative outcomes between the residual group and non-residual group, the postoperative UDVA was better in the non-residual group as of two weeks postoperatively. Because the residual group had a residual myopia greater than −0.75 D, which could reduce the visual acuity [18], it is reasonable that the postoperative UDVA was significantly better in the non-residual group than in the residual group. On the other hand, the UNVA did not reveal a significant difference between the residual and non-residual groups throughout the study period, except at the one-week postoperative point. At that point, the UNVA in the residual group was significantly better than in the non-residual group. Still, the mean postoperative UNVA difference between the two groups was about two lines on the near-vision chart, thus the residual group could have better near-visual acuity in real life [30]. The non-significant difference in UNVA between the two groups may results from the relatively high SD in both groups and the low patient numbers of our study population. The postoperative SEs were significantly higher in the residual group than in the non-residual group due to the grouping strategy in the present study. If we separate the sphere power and cylinder power, the mean postoperative sphere powers throughout the study period were −0.18 D and −0.89 D in the non-residual and residual groups, respectively. Thus, the amplitude of postoperative astigmatism may be acceptable in the two groups.

Comparing the visual and refractive outcomes of the non-residual group in the present study to previous research, the mean final postoperative UDVA in the non-residual group was 0.07. In the preceding study, the mean postoperative UDVA was about 0.05 to 0.13 in the non-diffractive EDOF IOL, and the postoperative UDVA in the non-residual group could be compatible to that EDOF IOL [10]. The mean postoperative UNVA was 0.24 in the EDOF group, which is similar to the mean UNVA in the preceding studies that implanted the EDOF IOL [31]. Regarding postoperative refraction, the mean final postoperative SE and sphere power were −0.21 D and −0.11 D, respectively, in the non-residual group, which did not exceed the minimal unit of refraction of −0.25 D, and may not have significant influence on visual acuity. In the previous study that implanted the toric IOL, the mean postoperative SE was −0.48 D, and the final postoperative SE in the non-residual group was comparable to the previous result [32]. Furthermore, the mean postoperative SE in the previous study that implanted the EDOF IOL was near −0.60 D, and the refractive results of the non-residual group were not inferior to the SE [33]. As a consequence, the visual and refractive results in the non-residual group could be comparable to those in the previous research concerning EDOF IOL implantation. However, the visual and refractive outcomes in the residual group were worse than in the non-residual group and the previous studies [10,31,32,33]. Accordingly, the importance of determining the risk factor for undercorrection in EDOF IOL implantation cannot be overemphasized.

There were a few limitations in the present study. Firstly, the retrospective design of the present study would diminish the homogeneity between the study populations. Secondly, the case numbers were low in the present study, with only 42 eyes included, and this could contribute to prominent statistical bias. In addition, the case numbers were discordant between the two groups, as the number of eyes in the non-residual group was five-fold that in the residual group. However, we already reduced the case number of non-residual cases in the matching process. Because the numbers of postoperative residual myopia were relatively low in our institution, a 1:1 or 1:2 ratio of residual and non-residual eyes could cause extreme difficulty for statistical analysis. Finally, we did not perform all the preoperative exams in the postoperative period in our routine practice. Accordingly, several factors like the postoperative biometric and topographic indexes cannot be accessed, which could decrease the integrity of our results.

## 5. Conclusions

In conclusion, high preoperative myopia and corneal refractive power correlated to a high chance of undercorrection following EDOF IOL implantation. Furthermore, the high-myopia population has more predisposing factors for undercorrection compared to the low-myopia population. Consequently, the strategy for IOL power selection might be modified in individuals scheduled for EDOF IOL implantation who present risk factors of undercorrection. Further large-scale prospective study to evaluate the optimal strategy for EDOF IOL power selection in individuals with risk factors of undercorrection is crucial.

## Figures and Tables

**Table 1:** The baseline features of the study population.

Feature	Non-Residual Group (*N*: 35)	Residual Group (*N*: 7)	*p*
Age (years, mean ± SD)	59.00 ± 9.98	56.20 ± 12.33	0.699
Sex (male:female)	15:20	2:5	0.580
Laterality (right:left)	12:23	4:3	0.343
Disease			0.085
Hypertension	4	2	
Diabetes mellitus	1	3	
Other	4	0	
Refractive surgery	3	1	0.545
UDVA (LogMAR)	0.49 ± 0.17	0.30 ± 0.25	0.112
CDVA (LogMAR)	0.37 ± 0.22	0.24 ± 0.14	0.240
Cycloplegia refraction (D)			
Sphere	−2.25 ± 5.43	−2.60 ± 5.36	0.438
Cylinder	−1.36 ± 0.83	−1.10 ± 0.89	0.518
SE	−2.93 ± 5.28	−3.05 ± 5.51	0.394
Topography			
TCRP	42.10 ± 2.99	44.62 ± 2.45	0.052
Cylinder power	1.01 ± 0.54	1.27 ± 0.66	0.597
CCT	529.77 ± 25.08	552.60 ± 32.82	0.190
Angle Kappa	0.21 ± 0.07	0.13 ± 0.08	0.147
Pupil diameter	3.76 ± 0.82	3.44 ± 0.36	0.581
Total HOA	0.30 ± 0.13	0.27 ± 0.17	0.463
SA	0.55 ± 0.55	0.58 ± 0.24	0.147
AXL	23.78 ± 1.26	25.28 ± 1.79	0.112
ACD	3.04 ± 0.43	3.27 ± 0.47	0.438
WTW	11.91 ± 0.21	12.04 ± 0.41	0.364
LT	4.58 ± 0.25	4.54 ± 0.62	0.774
ECD	2816.33 ± 308.40	3038.00 ± 288.17	0.082
CV	27.88 ± 2.71	29.80 ± 3.42	0.117
HEX	68.09 ± 4.63	62.82 ± 7.42	0.190
Femtosecond laser	6	2	0.610
Toric IOL	9	2	0.797

ACD: anterior chamber depth, AXL: axial length, CCT: central corneal thickness, CDVA: corrected distance visual acuity, CV: coefficient of variance, D: diopter, ECD: endothelial cell density, HEX: hexagonality, HOA: higher-order aberration, IOL: intraocular lens, LT: lens thickness, *N*: number, SA: spherical aberration, SD: standard deviation, SE: spherical equivalent, TCRP: total corneal refractive power, UDVA: uncorrected distance visual acuity, WTW: white-to-white.

**Table 2:** Postoperative visual and refractive conditions between the two groups.

Outcome	Non-Residual Group (*N*: 35)	Residual Group (*N*: 7)	*p*
UDVA			
1 day	0.08 ± 0.09	0.15 ± 0.21	0.099
1 week	0.10 ± 0.16	0.17 ± 0.21	0.138
2 weeks	0.08 ± 0.12	0.23 ± 0.26	0.017 *
1 month	0.07 ± 0.07	0.22 ± 0.19	0.010 *
UNVA			
1 day	0.28 ± 0.06	0.19 ± 0.18	0.053
1 week	0.34 ± 0.13	0.17 ± 0.27	0.036 *
2 weeks	0.25 ± 0.14	0.25 ± 0.18	0.797
1 month	0.24 ± 0.22	0.12 ± 0.08	0.125
SE			
1 day	−0.39 ± 0.30	−1.10 ± 0.64	0.004 *
1 week	−0.23 ± 0.26	−1.15 ± 0.68	0.001 *
2 weeks	−0.29 ± 0.27	−1.10 ± 0.68	0.002 *
1 month	−0.21 ± 0.35	−1.12 ± 0.57	<0.001 *

*N*: number, SE: spherical equivalent, UDVA: uncorrected distance visual acuity, UNVA: uncorrected near visual acuity. * denotes significant difference between groups.

**Table 3:** The risk factor for residual myopia in the whole population.

Factor	aOR	95% CI		*p*
		Lower	Upper	
Cycloplegia Sphere	2.315	1.495	4.061	<0.001 *
TCRP	1.624	1.158	2.417	0.001 *
Corneal cylinder	1.232	1.074	1.581	0.023 *
CCT	0.977	0.912	1.128	0.694
Pupil diameter	1.160	0.824	1.499	0.513
Total HOA	1.222	0.900	1.658	0.171
SA	0.943	0.782	1.167	0.205
Angle kappa	1.246	0.933	1.471	0.178
AXL	1.982	1.207	3.354	<0.001 *
ACD	1.378	0.989	1.546	0.082
WTW	1.007	0.865	1.387	0.729
LT	0.975	0.911	1.102	0.840

ACD: anterior chamber depth, aOR: adjusted odds ratio, AXL: axial length, CCT: central corneal thickness, CI: confidence interval, HOA: higher-order aberration, LT: lens thickness, SA: spherical aberration, TCRP: total corneal refractive power, WTW: white-to-white. * denotes significant correlation to residual myopia.

**Table 4:** The risk factor for residual myopia in the high-myopia population.

Factor	aOR	95% CI		*p*
		Lower	Upper	
Cycloplegia Sphere	3.527	2.459	5.198	<0.001 *
TCRP	2.287	1.642	3.114	<0.001 *
Corneal cylinder	1.673	1.398	1.989	<0.001 *
CCT	0.959	0.872	1.153	0.666
Pupil diameter	1.217	0.727	1.435	0.525
Total HOA	1.395	0.948	1.781	0.102
SA	1.006	0.926	1.208	0.158
Angle kappa	1.334	0.992	1.572	0.064
AXL	2.525	1.753	3.994	<0.001 *
ACD	1.523	1.174	1.965	<0.001 *
WTW	1.448	1.000	1.876	0.049 *
LT	1.042	0.937	1.189	0.706

ACD: anterior chamber depth, aOR: adjusted odds ratio, AXL: axial length, CCT: central corneal thickness, CI: confidence interval, HOA: higher-order aberration, LT: lens thickness, SA: spherical aberration, TCRP: total corneal refractive power, WTW: white-to-white. * denotes significant correlation to residual myopia.

**Table 5:** The risk factor for residual myopia in the low-myopia population.

Factor	aOR	95% CI		*p*
		Lower	Upper	
Cycloplegia Sphere	1.562	1.173	1.869	0.005 *
TCRP	1.340	1.124	1.615	0.022 *
Corneal cylinder	1.257	0.951	1.453	0.097
CCT	0.996	0.869	1.165	0.778
Pupil diameter	1.082	0.811	1.254	0.621
Total HOA	1.036	0.967	1.288	0.704
SA	0.955	0.841	1.168	0.638
Angle kappa	1.197	0.929	1.384	0.427
AXL	1.389	1.203	1.746	0.009 *
ACD	1.102	0.935	1.357	0.183
WTW	1.019	0.891	1.232	0.664
LT	0.914	0.832	1.077	0.786

ACD: anterior chamber depth, aOR: adjusted odds ratio, AXL: axial length, CCT: central corneal thickness, CI: confidence interval, HOA: higher-order aberration, LT: lens thickness, SA: spherical aberration, TCRP: total corneal refractive power, WTW: white-to-white. * denotes significant correlation to residual myopia.

## Data Availability

The data are available from the corresponding author upon reasonable request.
